# Proof-of-concept of real-time electromagnetic guidance for gynecologic interstitial catheters in high dose rate brachytherapy

**DOI:** 10.1016/j.phro.2024.100661

**Published:** 2024-10-24

**Authors:** Audrey Cantin, Marie-Claude Lavallée, Eric Poulin, Gilion Hautvast, William Foster, Luc Beaulieu

**Affiliations:** a Radiation Oncology, CHU de Québec – Université Laval, Québec, Canada; b EI Clinical Informatics, Philips, Eindhoven, the Netherlands; c CRCHU de Québec et Axe Oncologie, CHU de Québec – Université Laval, Québec, Canada; d Département de physique, génie physique et d’optique et Centre de recherche sur le cancer, Université Laval, Québec, Canada

**Keywords:** Brachytherapy, High Dose Rate, Cervical cancer, Electromagnetic tracking, Image guidance

## Abstract

**Background and Purpose:**

The addition of interstitial needles to intracavitary gynecologic (GYN) high dose rate (HDR) brachytherapy has been shown to improve target coverage and organs-at-risk (OAR) sparing. However, no commercial solution allows real-time guidance of interstitial catheter placement. This phantom study aimed to evaluate the feasibility of an electromagnetic (EM) tracking system guidance workflow for GYN HDR brachytherapy treatment in a magnetic resonance imaging (MRI) and real-time transrectal ultrasound (TRUS) fusion scenario.

**Materials and Methods:**

A clinical investigational system combining a treatment planning system and the EM tracking technology was used. The 3D T2 weighted magnetic resonance (MR) image set of a patient treated with intracavitary and interstitial HDR brachytherapy was retrospectively chosen. The MR image set was used to delineate the target and the OARs. A preplan was generated to determine needles positions in advance. The implant was reproduced in a water phantom. A 3D TRUS scan was acquired, and a rigid registration between the MR and the TRUS images was performed.

**Results:**

The accuracy of the EM tracking system was < 1 mm for both the sagittal and the transverse modes of the TRUS probe. Contours that were delineated on the MRI were propagated on the TRUS images after the rigid registration. Needle insertion was successfully guided in real time with the EM tracking system on the TRUS live image using the MRI contours for guidance.

**Conclusion:**

Based on this proof-of-concept, real-time EM-guidance of interstitial needle for GYN HDR brachytherapy appears to be feasible.

## Highlights

• Electromagnetic technology tracked needle insertion for gynecologic brachytherapy.
• Custom stylet enabled automatic gynecologic applicator reconstruction.
• Magnetic resonance imaging with ultrasound were combined to perform live guidance.
• Needles were guided on the ultrasound-electromagnetic tracking reference frame.

## 1 Introduction

In combination with chemotherapy and external beam therapy, high dose rate (HDR) brachytherapy has been shown to increase overall survival and tumor control for cervical cancer [1], [2]. A combination of interstitial and intracavitary brachytherapy results in a better target dose and organs at risk (OARs) sparing compared to intracavitary brachytherapy alone. The addition of needles helps to achieve better dose conformity to the tumor, particularly when parametrial extensions need to be treated [3], [4], [5]. These needles need to be carefully implanted to be useful and to allow dose escalation to the tumor volume. Many imaging modalities are available to visualize in three dimensions (3D) the patient anatomy. The retroEMBRACE study shows the advantage of image-guided HDR brachytherapy for local control and pelvic control for local cervix cancer [6].

Although the computed tomography (CT) imaging is well established for brachytherapy planning due to its accessibility and good bone-to-soft-tissue contrast, current guidelines recommend using magnetic resonance imaging (MRI) to implement 3D image-based gynecologic (GYN) HDR brachytherapy. MRI provides excellent soft-tissue contrast, improving tumor delineation in cervical cancer and visualization of surrounding organs [7], [8], [9], [10], [11]. These imaging modalities can be used in combination with the ultrasound (US) imaging, particularly useful for its real-time guidance.

The transabdominal and transrectal US (TRUS) imagings help guiding in real time applicator and interstitial needle placement, allowing dynamic adjustment during the brachytherapy procedure [12], [13]. However, it can be difficult to assess the depth and the exact position of the needle tip on the US image due to artifacts generated by the applicator itself, the limited field-of-view of TRUS and the presence of an anechoic or echogenic region in the patient anatomy [14]. Thus, a tracking tool is helpful to correctly localize in 3D the applicator and needles. The electromagnetic (EM) tracking technology is particularly suitable for brachytherapy, meeting real-time and localization criteria [15]. With this technology, positions and angulation of sensors within a magnetic field are known by the current induced in the sensors [16]. For instance, EM tracking is used with TRUS for prostate HDR brachytherapy. With this technology, it is possible to automatically track and reconstruct catheters, overcoming the issue of artifacts on TRUS images [17], [18], [19], [20].

The goal of this phantom study was to evaluate the feasibility of an EM tracking guidance workflow for interstitial catheter insertion in GYN HDR brachytherapy using an MRI and real-time TRUS fusion scenario. The hypothesis was that it was possible to use EM tracking in GYN brachytherapy to accurately guide in real time interstitial needles placement while taking advantage of a multi-modality imaging, combining the high soft-tissue contrast of MRI and the high accuracy guidance and reconstruction of EM tracking.

## 2 Materials and methods

### 2.1 System overview

The system used for this project was a clinical investigational system using EM tracking technology developed by *Philips Innovation & Strategy* and has already been presented [17], [18], [21]. This system was built around a workstation with a complete integrated treatment planning system (TPS) using AAPM TG43 dose calculation formalism [22]. It has been used in this project to perform contours on a pre-implant GYN magnetic resonance (MR) images and a live procedure was simulated in water by using the fusion module allowing rigid registration between MR-US and US-US images. Connected to the Flex Focus 400 (BK medical, 8848 biplanar probe), the navigation in the US image reference frame relative to the EM reference frame was enabled via a calibration process previously described in detail [21]. The TPS used an inverse-planning algorithm for needles positioning and dose optimization based on the MR contours. The optimization was used to perform planning both before (to carry out preplan on MRI) and during the brachytherapy intervention live on US.

In a previous study, it was shown that the accuracy of the EM tracking system was within 1 mm for both the sagittal and transverse modes of the US probe [21]. However, the stylet designed for prostate brachytherapy was too stiff to enter the applicator and the curved needles used around the ring applicator. Therefore, a new home-made stylet was designed based on a 5 degrees-of-freedom (5DOF) sensor from NDI (Aurora 5DOF sensor, P/N 610090) welded at the end of an optical fiber. The lead wires connected to the sensor were wrapped around the optic fiber. The latter was coated with a heatshrink-protecting tube to ensure its strength and flexibility and to allow its insertion in the Elekta interstitial needles and applicator (see Supplementary material Figure S1). The precise location of the sensor within this assembly in the EM tracking coordinate system was determined following a specific calibration protocol [17], [19]. Finally, the stylet was used within the Philips’ system where the US-to-EM coordinate system was validated by using a needle in water and localizing the tip of the needle for various positions on the US image with the stylet inserted: 9 positions for the sagittal transducer and 9 positions for the transverse transducer (comprising 3 different probe angles for both planes) [21]. After the calibration, needle tip reconstruction accuracy was assessed by measuring the residual length at the exit of the template front faceplate for 6 implanted needles in water and comparing it to the one predicted by the EM reconstructed path [21].

The template grid included in the system was designed for prostate brachytherapy and allowed predetermined needles positions, which were not compatible with the applicator and needles in this project. Thus, a new template with 250 holes of 2 mm diameter spaced 2 mm apart was (virtually) defined in the system. The template was then calibrated by inserting the stylet tip in the four corner holes of a template having the same physical dimensions, ensuring its usage in the US-to-EM coordinate system [17], [21].

### 2.2 Clinical case

For this phantom study, a patient with a cervical cancer treated with brachytherapy was retrospectively chosen. The implant was a combination of intracavitary and interstitial brachytherapy, consisting of the Elekta CT/MR ring applicator set (30 mm ring diameter and intra-uterine tube of 60 degrees and length of 40 mm) as well as 13 interstitial needles (9 ProGuide transvaginal needles in the guide hole of the ring and 4 additional transperineal needles). The treatment consisted of 4 fractions of 7 Gy, in 4 different insertions. The CT image was used for the planning, and the MRI (3D T2 weighed MR images, 1 mm isotropic resolution) was acquired at the first insertion with the implant in place and then registered to the CT image to help delineating the contours. OncentraBrachy v4.6 (Elekta Brachy, Veenendaal, The Netherlands) treatment planning system was used clinically, and the planning was performed with the inverse-planning algorithm IPSA. The treatment was delivered with the Elekta’s Flexitron afterloader.

### 2.3 Offline preplan

For this study, only the MR image set with ring applicator, intra-uterine tube and interstitial needles in place were imported into the Philips’ system to test the feasibility of the EM tracking. The MR image set was used to create an offline preplan in the Philips’ system. High-risk clinical target volume (HR-CTV) and OARs (consisting of the bladder and the rectum) were delineated on the MR images based on the EMBRACE II protocol [8]. Bowel and sigmoid were not used in this proof-of-concept, however these dose objectives could be added for clinical use. The offline preplan was generated, optimizing needles positioning and calculated dose distribution.

As the Philips’ system was designed for prostate brachytherapy, there was no applicator model in the embedded TPS. Hence, additional contours were created to mimic the applicator in order to use the inverse planning functionality of Philips’ TPS. Target was defined as the union of the applicator and the HR-CTV to ensure dose coverage at the surface of the ring applicator. Prescribed dose was 7 Gy to the target volume. Optimization criteria were similar to the objectives used in our clinic, respecting EMBRACE II recommendations. In particular, dose criteria were defined to ensure target coverage, to limit high dose in the tumor and to protect the OARs as detailed in Table 1. After a first optimization, manual plan modifications and the addition of needles were made to refine the newly created offline preplan.

**Table 1:** Dose objectives used for preplan dose distribution evaluation.

Contours		Dose objectives (prescribed dose = 7 Gy)
Target	HR_CTV	V100% > 95 % V150% < 45–55 % V200% < 20–30 %
OARs	Bladder	D2cm^3^ < 5.50 Gy
	Rectum	D2cm^3^ < 4.50 Gy

### 2.4 Water phantom live procedure and EM tracking

To simulate a live US procedure, the implant was reproduced in a water phantom with the ring applicator mounted on the TRUS stepper with the custom template consisting of non-metallic material to avoid any disturbance with the EM field (see Supplementary material Figure S2). It was designed to hold the applicator and to allow needle insertion as described previously. A 3D-US scan was acquired with the sagittal transducer by manually rotating the probe in water with the applicator in place. At this stage, the implant consisted of the ring applicator, the IU tube and the preselected transvaginal needles around the ring.

MRI-to-US rigid registration was performed with the Philips’ system fusion module using the IU tube and the ring applicator as reference objects. All the contours delineated on the MR images were then propagated on the US images. Finally, the preplan with the needles positions proposed by the TPS were transferred from the MR preplan to the joint US-EM reference frame. Needles were inserted according to the suggested preplan. Navigation was done by the EM tracking with the MRI contours superimposed on the live US images. The EM technology was used to track in real time the needles depth and position, and to automatically reconstruct the needles using the custom stylet. The latter was also inserted in the ring applicator and the IU tube to digitally reconstruct their inner channels and to allow their use in a live plan. That live plan was optimized taking into account the reconstructed final needles positions and applicator channels. HR-CTV coverage was evaluated with the needles already implanted and, if needed, transperineal needles can be added live to improve the dose distribution.

## 3 Results

The geometric measurements of the stylet sensor with the pivot calibration method placed the sensor at 5.1 mm (± 0.1 mm) of the stylet tip and was used in the system to enable accurate reconstruction of the channel. The calibration of the US-to-EM coordinate system with the custom stylet resulted in residual differences of 0.6 mm (± 0.2 mm) in the sagittal plane and 0.8 mm (± 0.2 mm) in the transverse plane, in agreement with prior results obtained by our team. The mean difference between residual lengths predicted by the EM reconstructed path and measured ones was 0.8 mm (± 0.3 mm).

**Fig. 1:**
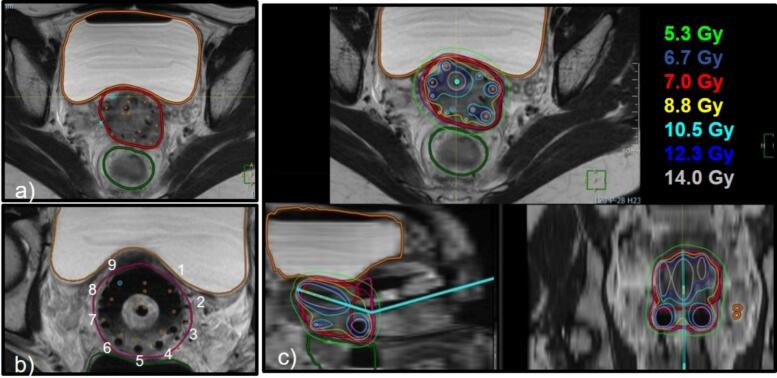
MRI preplan workflow. (a) The needles positions proposed by the TPS are shown in orange and superimposed on the MR images with the implant in place. (b) Proposed transvaginal needles are identified to the corresponding holes in the ring applicator (labeled 1 to 9 in the picture). (c) Manual adjustments are performed to fit the angulation of the IU tube and to fine-tune needles positions, and dose is optimized to ensure adequate target coverage and OARs sparing with the proposed implant.

Fig. 1 shows how the clinical investigational system was used to proceed to the preplan. The needles positions were optimized based on the HR-CTV contour (see Fig. 1a), and the proposed transvaginal needles were associated to corresponding holes in the ring applicator (see Fig. 1b). Needles were manually edited, and a needle was added to mimic the IU tube. All needles were available for dose optimization (Fig. 1c). For this particular case, all the positions in the ring template were chosen based on the offline preplan generated with the clinical investigational system, i.e. 9 needles, and 2 additional transperineal needles were added.

**Fig. 2:**
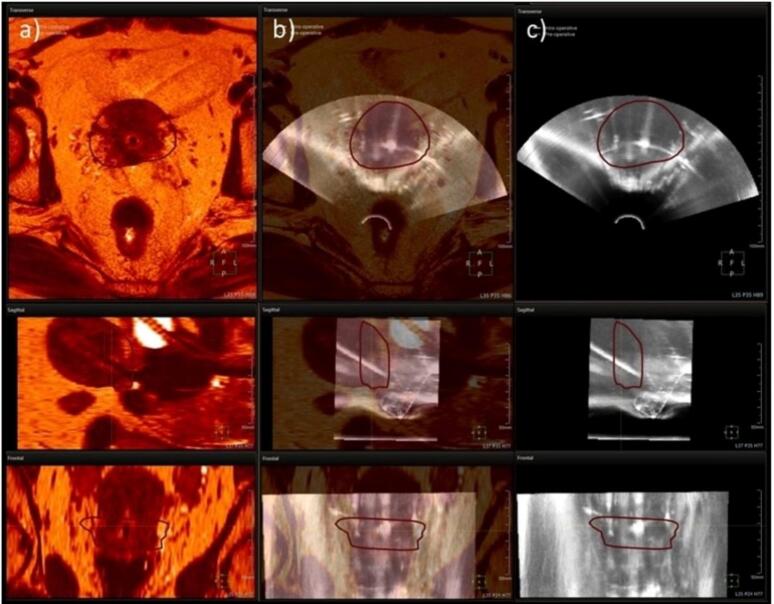
Axial, sagittal and frontal views of the (a) MR image set, (b) the MRI-US fusion and (c) the US image set. Registration is made with the TPS fusion tool and is based according to the ring applicator and the IU tube. Only the HR-CTV contour is shown.

Fig. 2 shows the outcome of the 3D US acquisition followed by rigid registration between the MR and US images. The target as well as the OARs contours from the MR were propagated on the US images after performing the rigid registration based on the applicator (Fig. 2). Navigation was performed by EM tracking with the MR contours superimposed on the US live image.

**Fig. 3:**
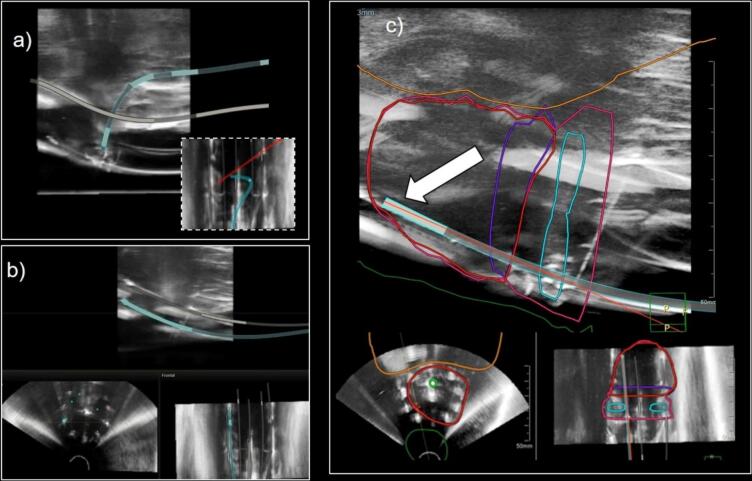
EM tracking in an MRI-US fusion scenario. US-to-EM reference frames calibration allow the EM navigation on the US. (a) The stylet is used to digitally reconstruct the applicator. The stylet path is lost halfway through the reconstruction of the inner channel of the ring, resulting in only half of the ring being reconstructed (as shown in the dotted-frame coronal view). (b) The 6 posterior transvaginal needles are reconstructed with the stylet, while the 3 anterior needles keep their preplanned positions until reconstruction. Those reconstructions are displayed in all three perpendicular views and fit on the US image. (c) The MRI contours are superimposed on the US image. Live tracking of the stylet inserted in the needle is shown by the red line (indicated by the white arrow), allowing to finely adjust the needles depth based on the target contour.

The stylet was inserted in the IU tube, allowing to digitally reconstruct its inner channel and the ring. However, the path of the ring was lost halfway through the reconstruction resulting in only half of the ring being reconstructed (Fig. 3a). Depths of the transvaginal needles around the ring were adjusted in real time with the EM tracking and the MRI contours visible on the live US image as shown in Fig. 3c. The needles and the applicator reconstruction overlayed well with the visual provided by US image (see Fig. 3b).

## 4 Discussion

In this proof of concept, it was shown that interstitial GYN brachytherapy benefits from EM tracking technology. The high soft-tissue contrast of the MRI was used for contouring and for the creation of a preplan, while the US was used for its live image capability. EM navigating was thus performed in the live US image with the MRI contours overlayed. Artifacts affecting needles visibility on the live US were overcome by the EM tracking.

At the time of the ring insertion, the ProGuide guiding tubes need to be already attached to the applicator for the insertion of the transvaginal needles. The preplan helps to decide which of those needles are necessary to achieve an adequate dose distribution, and the EM tracking helps to guide in real time the trajectory and the depth of each needle. In this project, the preplan was generated using the inverse-planning module of the clinical investigational system. However, different optimizations could have been employed as alternative methods for preplanning [10]. The dose distribution is updated live with the MRI contours superimposed on the US images as the applicator and needles were automatically reconstructed from the retraction of the EM stylet.

Based on these results, a workflow is proposed. Prior to the intervention, the patient undergoes MR imaging with the applicator in place. The image set is imported in the TPS to proceed with the contours. A preplan is generated to define the transvaginal needles positions and to proceed with a first dose distribution optimization. At this step, it can also be determined if transperineal needles will be needed to cover the HR-CTV extensions. On the treatment day, the applicator with the appropriate guiding tubes and IU tube are inserted in the operating room. TRUS probe is used to acquire a US 3D image. The MRI-to-US registration is then made based on the applicator position and MRI contours are propagated on the US images. Needles selected from the preplan are inserted, and their depths are guided with EM tracking. If needed, transperineal needles can be added and their insertions also guided by EM tracking. In a multi-implant scenario, the MRI from the first insertion could be used to create the preplan to guide the subsequent fractions, thus eliminating the need for an MRI before the implant.

The investigational system used in this project was designed for prostate brachytherapy. It was used in this study for its treatment planning system allowing MRI-to-US registration while integrating EM tracking information. However, as seen in Fig. 3, there are limitations associated with its direct use for GYN procedures, namely its inability to fully reconstruct a channel path that loops back on itself i.e. a GYN ring applicator. This is due to the expected geometry of interstitial catheter in prostate HDR brachytherapy, simplifying the reconstruction algorithm currently implemented in the system. However, the fusion of MR-to-US based on the intra-uterine channel and half of the ring is enough to complete the necessary registration (as per the methodology described in [23]) and to fully demonstrate our proof-of-concept for guidance of interstitial catheter insertion in GYN HDR brachytherapy. For clinical use, a modification to the reconstruction algorithm would be necessary to allow for a final dose optimization. It should be pointed out that the accurate reconstruction of curved or loop paths using EM tracking was extensively demonstrated in a recent study [24]. To enable direct reconstruction of the ring applicator, the current system would need to be modified to incorporate a filtering based on the known geometry of the applicator, instead of the current back-folding prevention and polynomial filtering applied for catheters.

Second, the needle placement optimized by the TPS at the preplan stage (prior to EM tracking reconstruction) can only be along a parallel pattern confined to a template link to a TRUS probe e.g. a Syed-Neblett, a Venezia template and others). This pattern does not currently accommodate freehand needle insertion sometimes needed for interstitial GYN brachytherapy (although these needles can be added on the spot during the intervention and guided with the EM tracking). Finally, in this proof of concept, the deformation due to the TRUS probe is not considered. Possible solutions are performing the preplan MRI with the applicator and a dummy TRUS probe in place or using a deformation algorithm.

There are many tools available, whether in a clinical or research context, to enhance the quality of GYN brachytherapy treatments. For example, as shown in this project, it is possible to optimize needles placement in advance based on organ contours. This enables preplanning and potentially the reduction of the number of needles without compromising the plan quality, resulting in a less invasive procedure [25], [26]. Also, EM tracking technology can be employed as quality assurance before treatment to detect potential errors [23], [27], [28]. Furthermore, knowing the precise location of the tip of the needle in real time reduces the risk of insertion within an OAR. Moreover, the benefits of EM tracking could be more significant for recurrent tumors, for instance for vaginal recurrence of endometrial or cervical cancer, where precise needles placement plays a crucial role in achieving a positive outcome [29].

In conclusion, the feasibility of accurately guiding interstitial needle insertion for GYN HDR brachytherapy using EM tracking was demonstrated. Although the prototype system used in this project was not tailored for GYN brachytherapy, it was possible to combine MRI information with real-time US to perform both preplanning and live guidance. EM tracking technology allows real-time guidance of the position and depth of each needle as well as the automated reconstruction of each channel.

## Declaration of competing interest

The authors declare the following financial interests/personal relationships which may be considered as potential competing interests: This project was made possible in part via a research agreement with Philips Innovation & Strategy.
