# Supplementary material for: Proof-of-concept of real-time electromagnetic guidance for gynecologic interstitial catheters in high dose rate brachytherapy
Source: Phys Imaging Radiat Oncol. 2024 Oct 24;32:100661. doi: 10.1016/j.phro.2024.100661 (PMC11567093; doi:10.1016/j.phro.2024.100661)
Supplement: Supplementary Data 1 [file mmc1.pdf]

# Supplementary material

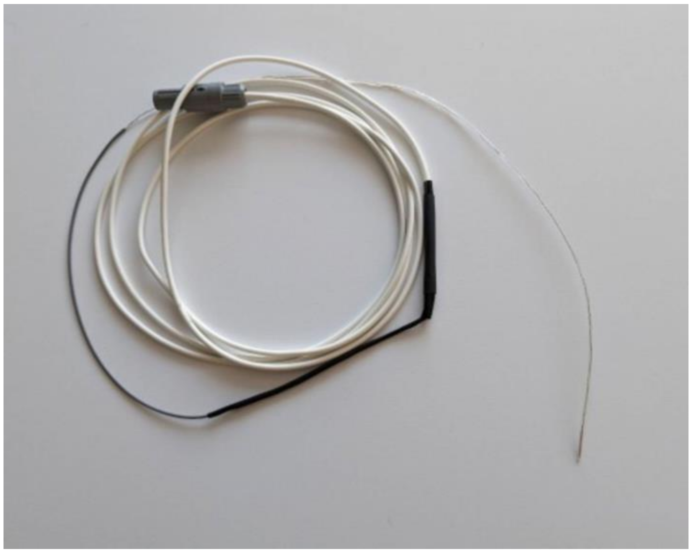

*Figure S1: Custom stylet consisting of a 5DOF sensor mounted on optic fiber and protected by a heatshrink-protecting tube.*

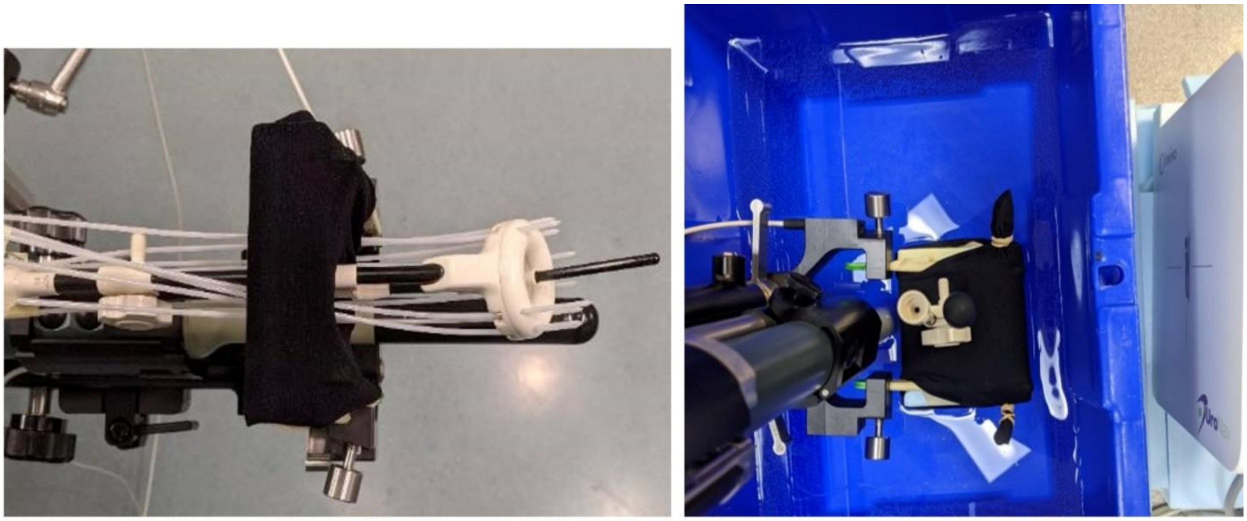

a) b)

*Figure S2: (a) The implant (consisting of the transvaginal needles and the applicator) and the TRUS probe are mounted on the stepper with the custom template to proceed with the US live procedure. (b) The implant is immersed in water to acquire a 3D TRUS scan. EM field generator of the clinical investigational system (white box) is positioned to allow EM navigation in the treatment area.*
